# Serum triglyceride glucose index is a valuable predictor for visceral obesity in patients with type 2 diabetes: a cross-sectional study

**DOI:** 10.1186/s12933-023-01834-3

**Published:** 2023-04-29

**Authors:** Qing Yang, Huichao Xu, Hongli Zhang, Yanying Li, Shuxiong Chen, Dongye He, Guangzhi Yang, Bo Ban, Mei Zhang, Fupeng Liu

**Affiliations:** 1 Department of Clinical Nutrition, Affiliated Hospital of Jining Medical University, Jining Medical University, Jining, China; 2 Department of Endocrinology, Affiliated Hospital of Jining Medical University, Jining Medical University, Jining, China; 3 Chinese Research Center for Behavior Medicine in Growth and Development, Jining, China; 4 Department of Medical Imaging, Affiliated Hospital of Jining Medical University, Jining Medical University, Jining, China; 5 Medical Research Centre, Affiliated Hospital of Jining Medical University, Jining, Shandong Province China

**Keywords:** Visceral obesity, Triglyceride glucose index, Type 2 diabetes mellitus

## Abstract

**Background:**

Since the triglyceride glucose (TyG) index can reflect insulin resistance, it has been proven to be an efficient predictor of glycolipid-metabolism-related diseases. Therefore, this study aimed to investigate the predictive value of the TyG index for visceral obesity (VO) and body fat distribution in patients with type 2 diabetes mellitus (T2DM).

**Methods:**

Abdominal adipose tissue characteristics in patients with T2DM, including visceral adipose area (VAA), subcutaneous adipose area (SAA), VAA-to-SAA ratio (VSR), visceral adipose density (VAD), and subcutaneous adipose density (SAD), were obtained through analyses of computed tomography images at the lumbar 2/3 level. VO was diagnosed according to the VAA (> 142 cm^2^ for males and > 115 cm^2^ for females). Logistic regression was performed to identify independent factors of VO, and receiver operating characteristic (ROC) curves were used to compare the diagnostic performance according to the area under the ROC curve (AUC).

**Results:**

A total of 976 patients were included in this study. VO patients showed significantly higher TyG values than non-VO patients in males (9.74 vs. 8.88) and females (9.59 vs. 9.01). The TyG index showed significant positive correlations with VAA, SAA, and VSR and negative correlations with VAD and SAD. The TyG index was an independent factor for VO in both males (odds ratio [OR] = 2.997) and females (OR = 2.233). The TyG index ranked second to body mass index (BMI) for predicting VO in male (AUC = 0.770) and female patients (AUC = 0.720). Patients with higher BMI and TyG index values showed a significantly higher risk of VO than the other patients. TyG-BMI, the combination index of TyG and BMI, showed significantly higher predictive power than BMI for VO in male patients (AUC = 0.879 and 0.835, respectively) but showed no significance when compared with BMI in female patients (AUC = 0.865 and 0.835, respectively).

**Conclusions:**

. TyG is a comprehensive indicator of adipose volume, density, and distribution in patients with T2DM and is a valuable predictor for VO in combination with anthropometric indices, such as BMI.

**Supplementary Information:**

The online version contains supplementary material available at 10.1186/s12933-023-01834-3.

## Introduction

Type 2 diabetes mellitus (T2DM), a complex endocrine-metabolic disease driven by a chronic positive energy balance, has become a major global public health crisis globally [1]. From 2000 to 2021, there was a 2.5-fold increase in the prevalence of T2DM worldwide, which is largely attributed to the obesity epidemic [2, 3]. Obesity, especially visceral obesity (VO), is an independent risk factor for T2DM, its complications and even all-cause mortality [4, 5].

Although body mass index (BMI) might be a convenient and simple index to monitor the growth in obesity prevalence at the population level, studies have shown that obesity defined by BMI cannot account for the extreme variation in intra-visceral fat distribution between individuals [6]. Furthermore, excessive visceral adipose tissue (VAT) accumulation is associated with endocrine system dysfunction and pro-inflammatory activity, which may contribute to the deterioration of insulin resistance and the development of vascular complications [7–10]. In contrast, subcutaneous adipose tissue (SAT) is reported as a weaker indicator of metabolic diseases [6, 11]. Therefore, an accurate assessment of the fat type is integral to understanding obesity-related comorbidities and the treatment of obesity.

Computed tomography (CT) is considered the gold standard for independently assessing visceral and subcutaneous abdominal fat mass [12]. In addition, CT can also evaluate fat density using radiographic pixels denoted in Hounsfield units (HU) and referred to as attenuation. A study evaluated 1829 Framingham Heart Study participants and found that lower abdominal fat density (more negative CT fat attenuation) is associated with adverse cardiometabolic risk [13]. However, because this method is expensive and requires radiation exposure, it is unsuitable for routine clinical practice in the general population [14].

The triglyceride glucose (TyG) index, which is calculated using triglyceride (TG) and fasting blood glucose (FBG), has become an attractive option for predicting insulin resistance [15]. It was first used by Simental-Mendia et al. 2008 [16]. Several recent studies have indicated that the TyG index is associated with inflammation, endothelial dysfunction, glucolipid metabolism disorders, thrombosis, and atherosclerotic disease[17–19]. Notably, the TyG index-related parameters, such as TyG-BMI, TyG-waist circumference (WC), and TyG-waist hip rate (WHR), showed higher predictive power for non-alcoholic fatty liver disease in Asians [20, 21]. Unfortunately, the correlation between the TyG index and VO, which is the main cause of insulin resistance in T2DM patients, remains unknown.

Therefore, in this study, we aimed to investigate whether the TyG index can adequately assess the fat distribution and predict VO in patients with T2DM.

## Methods

### Study population

T2DM patients hospitalized in the Department of Endocrinology, Affiliated Hospital of Jining Medical University, From January 2017–December 2021, were included in this study. All patients underwent CT scans, including the center plane of the third lumbar vertebra on axial images and blood analyses to determine FBG and TG levels. The exclusion criteria were as follows: (1) missing the major anthropometric measures, such as height and weight; (2) patients with malignant tumors, hepatic damage, immune disease, abnormal thyroid function, or stage V diabetic nephropathy; and (3) patients with acute complications or infections. On admission, all patients were informed that their medical records could be used for research unless they indicated their opposition. In the present study, none of the patients indicated opposition. This study was approved by the Ethics Committee of the Affiliated Hospital of Jining Medical University (No. 2021C041).

### Laboratory and anthropometric measurements

All biochemical and immune indices in the present study were measured at our hospital laboratory. FBG and TG levels were measured using an automatic analyzer (Cobas8000; Roche Diagnostics Ltd., Basel, Switzerland). The TyG index was calculated as the natural logarithm (Ln) of [TG (mg/dl) × FBG (mg/dl)/2]. The TyG-BMI, TyG-WC and TyG-WHR were calculated as TyG multiplied by BMI, WC and WHR, respectively. Both systolic blood pressure (SBP) and diastolic blood pressure (DBP) were measured using a sphygmomanometer after at least 5 min of seated rest. Body mass index (BMI) was calculated by dividing the body weight in kilograms by height in meters squared.

### Measurement of abdominal adipose fat area

Abdominal CT was performed using a Dual-Source Flash CT scanner (Siemens, Erlangen, Germany). Two authors identified axial CT images at the L3 level, where the spinous and two transverse processes could be visualized. Abdominal adipose fat was assessed using abdominal axial CT images at the L3 level and Slice-O-Matic software (V.5.0, TomoVision, Montreal, Quebec, Canada), as described in our previous study [14]. The CT attenuation thresholds ranged from − 150 to -30 HU for VAT and from − 190 to -30 HU for SAT [22]. The visceral adipose area (VAA), subcutaneous adipose area (SAA), visceral adipose density (VAD), and subcutaneous adipose density (SAD) were automatically calculated by the software and shown as square centimeter and the mean radiation attenuation of adipose tissue in HU. VO was diagnosed according to Huo’s criteria: a VAA > 142 cm^2^ for men and > 115 cm^2^ for women at the L2/3 level [14, 23]. The VAA-to-SAA ratio (VSR) was calculated by dividing the area of VAT by that of SAT.

### Definitions and diagnosis

Hypertension was defined as systolic blood pressure ≥ 140 mmHg, diastolic blood pressure ≥ 90 mmHg, and the use of antihypertensive medications. Dyslipidemia was defined as a disorder of lipoprotein metabolism and the use of lipid medications. Alcohol intake was defined as the consumption of at least 30 g of alcohol per week for at least a year. Cigarette smoking was defined as smoking at least 100 cigarettes in a lifetime [24].

### Statistical analysis

Continuous variables are summarized using the median and interquartile range if not normally distributed and the mean ± SD if normally distributed. Categorical variables were summarized as frequency counts and percentages. These variables were compared between the two groups using an independent samples t-test or Mann–Whitney U test. Categorical variables were analyzed using the χ2 test. Univariable correlations between variables were assessed using Pearson’s correlation coefficients. Variables with significant differences between the VO and non-VO groups were enrolled in the logistic regression analysis to identify independent factors of VO. Receiver operating characteristic (ROC) curve analyses were used to compare the diagnostic performance of the independent factors for VO according to the area under the ROC curve (AUC). The overall predictive power for VO was further analyzed by comparing their AUCs using the Z test [25]. Since WC and WHR were extracted from only a limited number of patients, their correlations with VAA and diagnostic performance for VO were presented as additional analysis. The Statistical analysis was performed using the SPSS software V.25.0 (IBMCorp., Armonk, N.Y., USA). A two-tailed statistical measure with a p-value less than 0.05 was considered significant.

## Results

### Clinical characteristics of enrolled patients

A total of 976 patients (614 men and 362 women) were enrolled in this study. Their adipose tissue and clinical characteristics are shown in Tables 1 and 2. There was an obvious difference in the adipose tissue phenotype between male and female patients. Male patients showed a higher level of VAA (168.14 cm^2^ vs. 116.10 cm^2^) but a lower level of SAA (131.05 cm^2^ vs. 153.51 cm^2^) than female patients.

**Table 1:** Adipose tissue and clinical characteristics of included male patients

Variables	Total male (n = 614)	Non-VO group (n = 243)	VO group (n = 371)	p value
Age (years)	53.01 ± 11.91	53.44 ± 12.18	52.73 ± 11.73	0.464
DM duration (years)	7.93 ± 6.86	8.40 ± 7.34	7.61 ± 6.52	0.174
BMI (kg/m^2^)	26.05 ± 3.62	23.65 ± 2.58	27.62 ± 3.33	< 0.001
SBP (mmHg)	135.50 ± 17.41	132.19 ± 16.86	137.6 8 ± 17.44	0.564
DBP (mmHg)	82.35 ± 13.1	79.19 ± 11.68	84.42 ± 13.58	< 0.001
Hemoglobin (g/L)	146.20 ± 19.40	142.31 ± 22.85	148.77 ± 16.26	< 0.001
Creatinine (µmol/L)	66.35 ± 15.86	64.01 ± 15.13	67.91 ± 16.17	0.003
Blood urea nitrogen (mmol/L)	5.96 ± 3.84	5.93 ± 3.60	5.98 ± 4.00	0.888
TG (mmol/L)	1.51 (1.00,2.43)	1.02 (0.76,1.39)	1.92 (1.39,3.19)	< 0.001
Total cholesterol (mmol/L)	4.57 ± 1.61	4.26 ± 1.09	4.78 ± 1.84	< 0.001
HDL (mmol/L)	1.13 ± 0.40	1.19 ± 0.37	1.08 ± 0.41	< 0.001
LDL (mmol/L)	2.67 ± 1.18	2.60 ± 0.84	2.71 ± 1.36	0.188
Albumin (g/L)	43.88 ± 4.56	43.17 ± 5.00	44.34 ± 4.17	0.003
Alanine transaminase (U/L)	19.40 (14.10,30.20)	17.40 (13.00,24.15)	21.40 (15.20,35.90)	< 0.001
FT3 (pmol/L)	4.59 ± 1.34	4.58 ± 1.49	4.61 ± 1.23	0.302
FT4 (pmol/L)	17.25 ± 7.43	17.25 ± 7.16	17.25 ± 7.61	0.962
TSH (pmol/L)	2.23 ± 1.72	2.02 ± 1.35	2.37 ± 1.92	0.180
HbA1c (%)	8.84 ± 2.18	8.73 ± 2.40	8.92 ± 2.03	0.335
FBG (mmol/L)	9.57 ± 3.96	8.99 ± 4.07	9.95 ± 3.85	0.004
TyG index	9.40 ± 0.96	8.88 ± 0.80	9.74 ± 0.91	< 0.001
VAA (cm^2^)	168.14 ± 82.77	89.85 ± 34.96	219.43 ± 62.36	< 0.001
SAA (cm^2^)	131.05 ± 59.61	100.08 ± 44.02	151.34 ± 59.81	< 0.001
VSR	1.34 ± 0.60	0.96 ± 0.40	1.59 ± 0.58	< 0.001
VAD (HU)	-90.03 ± 8.90	-82.64 ± 8.97	-94.88 ± 4.39	< 0.001
SAD (HU)	-92.81 ± 7.79	-89.07 ± 9.55	-95.26 ± 5.07	< 0.001
Cigarette smoking (%)	374 (60.9)	144 (59.3)	230 (62.0)	0.500
Alcohol intake (%)	418 (68.1)	158 (65.0)	260 (70.1)	0.215
Dyslipidemia (%)	353 (57.5)	89 (36.6)	264 (71.3)	< 0.001
Hypertension (%)	291 (47.4)	97 (39.9)	194 (52.3)	0.003
Antidiabetic drugs (%)	513 (83.6)	202 (83.1)	311 (83.8)	0.825
SGLT-2 inhibitors (%)	35 (5.7)	13 (5.3)	22 (5.9)	0.860
GLP-1RAs (%)	1 (0.2)	0 (0.0)	1 (0.3)	1.000
Insulin (%)	217 (35.3)	94 (38.7)	123 (33.2)	0.168
Thiazolidinediones (%)	39 (6.4)	15 (6.2)	24 (6.5)	1.000
Statin drugs (%)	114 (18.6)	41 (16.9)	73 (19.7)	0.397
Fibrates (%)	11 (1.8)	4 (1.7)	7 (1.9)	1.000
Antihypertensive drugs (%)	215 (35.1)	57 (23.5)	158 (42.8)	< 0.001

Abreviations: DM duration, diabetes mellitus duration; SBP, Systolic blood pressure; DBP, Diastolic blood pressure; HDL, high-density lipoproteins; LDL, low-density lipoproteins; FT3, free triiodothyronine; FT4, free thyroxine; TSH, thyroid-stimulating hormone; HbA1c, glycated hemoglobin; SGLT-2 inhibitors, sodium-glucose transport protein-2 inhibitors; GLP-1RAs, glucagon like peptide-1 receptor agonists

**Table 2:** Adipose tissue and clinical characteristics of included female patients

Variables	Total female (n = 362)	Non-VO group (n = 196)	VO group (n = 166)	p value
Age (years)	58.81 ± 10.21	57.19 ± 10.61	60.72 ± 9.38	0.001
DM duration (years)	9.76 ± 7.39	9.67 ± 7.54	9.87 ± 7.23	0.803
BMI (kg/m^2^)	24.88 ± 3.59	23.08 ± 2.77	27.01 ± 3.29	< 0.001
SBP (mmHg)	136.34 ± 21.01	132.29 ± 20.81	141.13 ± 20.29	< 0.001
DBP (mmHg)	77.21 ± 12.09	74.95 ± 11.29	79.87 ± 12.49	< 0.001
Hemoglobin (g/L)	127.60 ± 21.31	124.96 ± 22.86	130.70 ± 18.93	0.011
Creatinine (µmol/L)	50.88 ± 13.52	48.81 ± 13.13	53.29 ± 13.62	0.002
Blood urea nitrogen (mmol/L)	5.52 ± 6.28	5.74 ± 8.48	5.27 ± 1.29	0.479
TG (mmol/L)	1.37 (0.96,2.00)	1.14 (0.81,1.57)	1.83 (1.27,2.66)	< 0.001
Total cholesterol (mmol/L)	4.91 ± 1.21	4.87 ± 1.21	4.96 ± 1.21	0.520
HDL (mmol/L)	1.24 ± 0.28	1.30 ± 0.29	1.16 ± 0.25	< 0.001
LDL (mmol/L)	2.95 ± 0.91	2.94 ± 0.86	2.95 ± 0.96	0.905
Albumin (g/L)	42.84 ± 4.58	42.55 ± 4.62	43.18 ± 4.52	0.199
Alanine transaminase (U/L)	16.15 (11.90,24.68)	15.50 (11.10,20.38)	16.90 (12.50,27.78)	0.016
FT3 (pmol/L)	4.21 ± 1.18	4.19 ± 1.21	4.23 ± 1.15	0.746
FT4 (pmol/L)	15.61 ± 3.25	15.49 ± 3.28	15.75 ± 3.22	0.455
TSH (pmol/L)	3.01 ± 3.83	3.01 ± 4.49	3.02 ± 2.87	0.991
HbA1c (%)	9.21 ± 2.24	9.15 ± 2.32	9.29 ± 2.13	0.569
FBG (mmol/L)	10.05 ± 4.33	9.57 ± 4.36	10.61 ± 4.25	0.023
TyG index	9.27 ± 0.78	9.01 ± 0.71	9.59 ± 0.73	< 0.001
VAA (cm^2^)	116.10 ± 57.73	74.08 ± 27.15	165.72 ± 42.98	< 0.001
SAA (cm^2^)	153.51 ± 60.79	129.17 ± 46.99	182.24 ± 62.79	< 0.001
VSR	0.80 ± 0.41	0.63 ± 0.31	1.01 ± 0.41	< 0.001
VAD (HU)	-89.86 ± 8.60	-85.39 ± 8.02	-95.13 ± 5.86	< 0.001
SAD (HU)	-97.6 ± 6.20	-95.95 ± 6.39	-99.56 ± 5.38	< 0.001
Cigarette smoking (%)	8 (2.2)	4 (2.0)	4 (2.4)	1.000
Alcohol intake (%)	3 (0.8)	2 (1.0)	1 (0.6)	1.000
Dyslipidemia (%)	210 (58.2)	93 (47.7)	117 (70.5)	< 0.001
Hypertension (%)	165 (45.6)	69 (35.2)	96 (57.8)	< 0.001
Antidiabetic drugs (%)	333 (92.0)	179 (91.3)	154 (92.8)	0.699
SGLT-2 inhibitors (%)	20 (5.5)	8 (4.1)	12 (7.2)	0.249
GLP-1RAs (%)	1 (0.3)	0 (0)	1 (0.6)	0.459
Insulin (%)	173 (47.8)	93 (47.4)	80 (48.2)	0.916
Thiazolidinediones (%)	31 (8.6)	15 (7.7)	16 (9.6)	0.573
Statin drugs (%)	78 (21.6)	35 (17.9)	43 (25.9)	0.073
Fibrates (%)	9 (2.5)	4 (2.0)	5 (3.0)	0.738
Antihypertensive drugs (%)	127 (35.1)	47 (24.0)	80 (48.2)	< 0.001

Abreviations: DM duration, diabetes mellitus duration; SBP, Systolic blood pressure; DBP, Diastolic blood pressure; HDL, high-density lipoproteins; LDL, low-density lipoproteins; FT3, free triiodothyronine; FT4, free thyroxine; TSH, thyroid-stimulating hormone; HbA1c, glycated hemoglobin; SGLT-2 inhibitors, sodium-glucose transport protein-2 inhibitors; GLP-1RAs, glucagon like peptide-1 receptor agonists

**Fig. 1:**
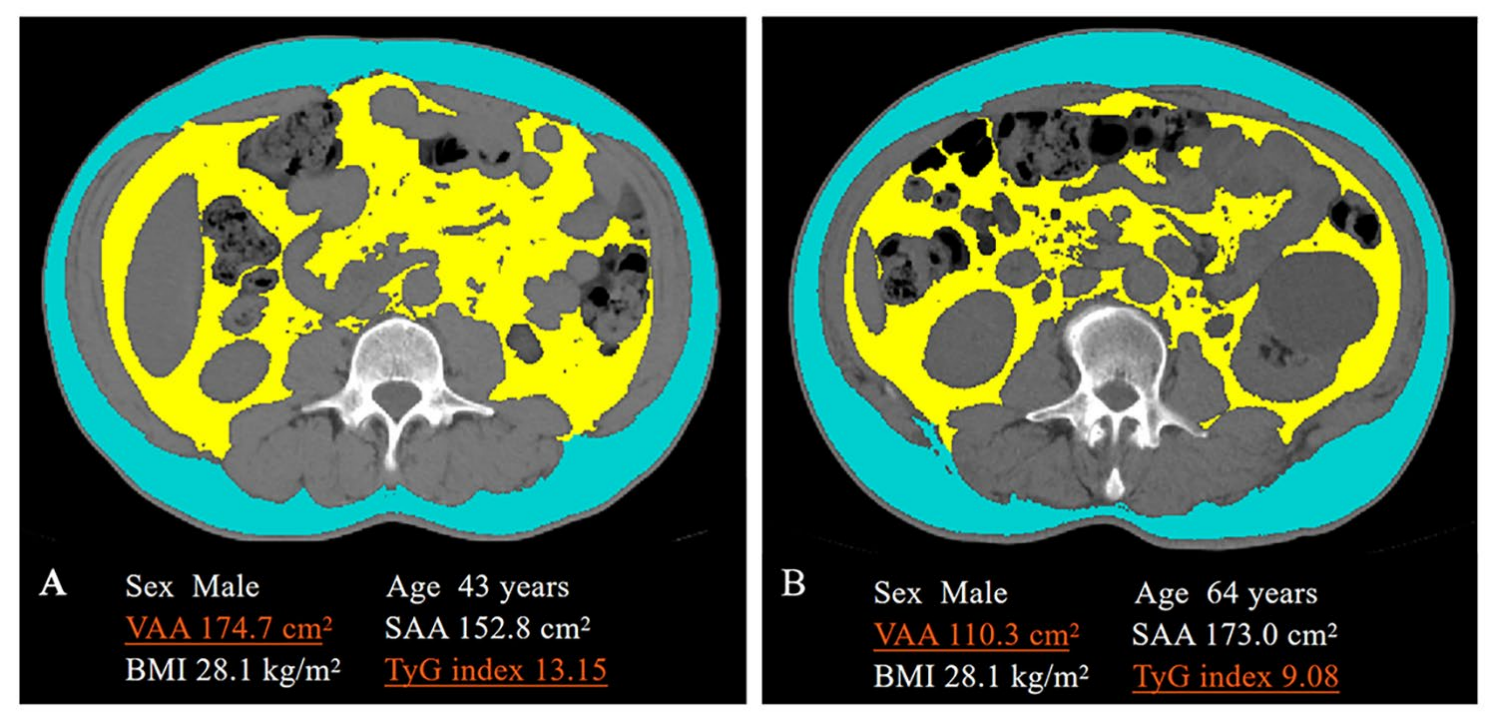
CT image analysis at the lumbar 3 level using Slice-O-Matic software. Blue: subcutaneous adipose tissue; yellow: visceral adipose tissue; grey: unprocessed CT image

Except for higher areas of visceral and subcutaneous adipose tissues, VO patients showed higher levels of VSR and lower levels of VAD and SAD. Regarding other clinical features, VO patients showed higher BMI, SBP, DBP, hemoglobin, creatinine, alanine transaminase, TG, FBG and TyG index levels and higher percentages of dyslipidemia and hypertension. On an individual level, patients with the same BMI may have a different level of VAA. As shown in Fig. 1, patient A had a similar BMI to patient B but much higher levels of VAA.

### Correlations between adipose tissue and clinical characteristics

**Fig. 2:**
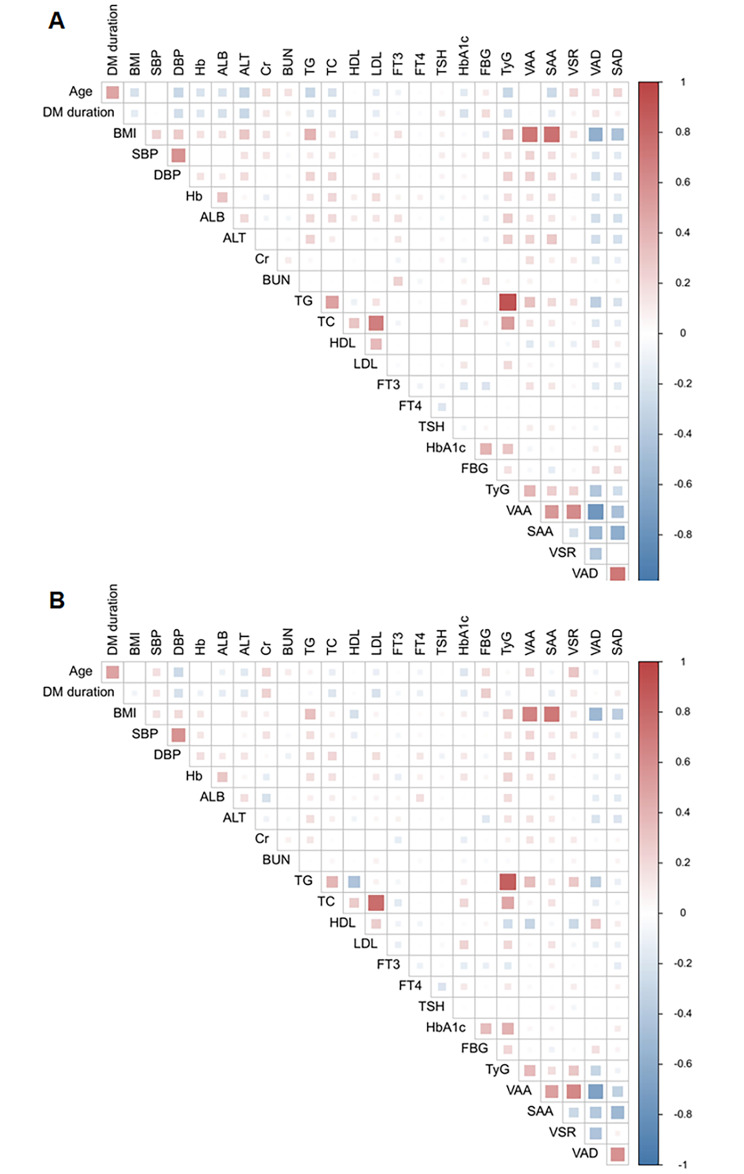
Correlations between adipose tissue and clinical characteristics. **(A)** male patients; **(B)** female patients. Abreviations: SBP, Systolic blood pressure; DBP, Diastolic blood pressure; Hb, hemoglobin; ALB, Albumin; ALT, alanine transaminase; Cr, creatinine; BUN, Blood urea nitrogen; TG, triglyceride; TC, Total cholesterol; HDL, high-density lipoproteins; LDL, low-density lipoproteins; FT3, free triiodothyronine; FT4, free thyroxine; TSH, thyroid-stimulating hormone; HbA1c, glycated hemoglobin; FBG, fasting blood glucose; TyG, triglyceride glucose index; VAA, visceral adipose area; SAA, subcutaneous adipose area; VSR, VAA-to-SAA ratio; VAD, visceral adipose density; SAD, subcutaneous adipose density. ALT and TG were log-transformed in the analysis

The correlations between adipose tissue and other clinical characteristics are shown in Fig. 2. In males, the TyG index was significantly correlated with VAA (r = 0.380), SAA (r = 0.258), VAD (r =-0.403), and SAD (r =-0.249). In females, the TyG index showed similar significant correlations with VAA (r = 0.365), SAA (r = 0.172), and VAD (r =-0.296) but was not significantly correlated with SAD (r =-0.084). Generally, the TyG index ranked second only to BMI on the degree of correlation with the above adipose indices. It is worth noting that the TyG index showed a stronger correlation with VSR than BMI in both males (r = 0.228 vs. 0.137) and females (r = 0.296 vs. 0.111).

### Predictive ability of TyG index

**Fig. 3:**
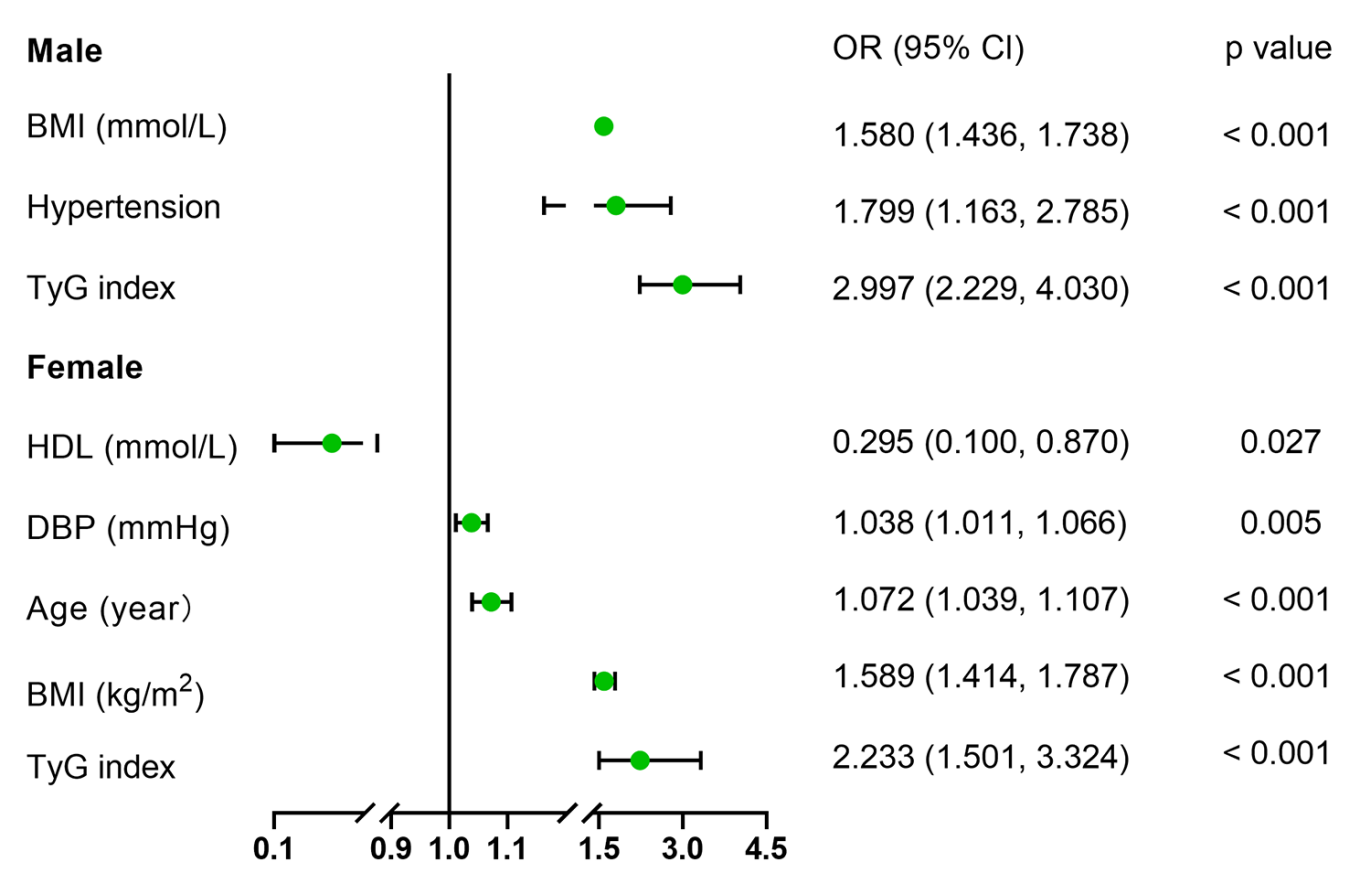
Forest plot of independent factors identified from logistic linear regression for VO.

**Fig. 4:**
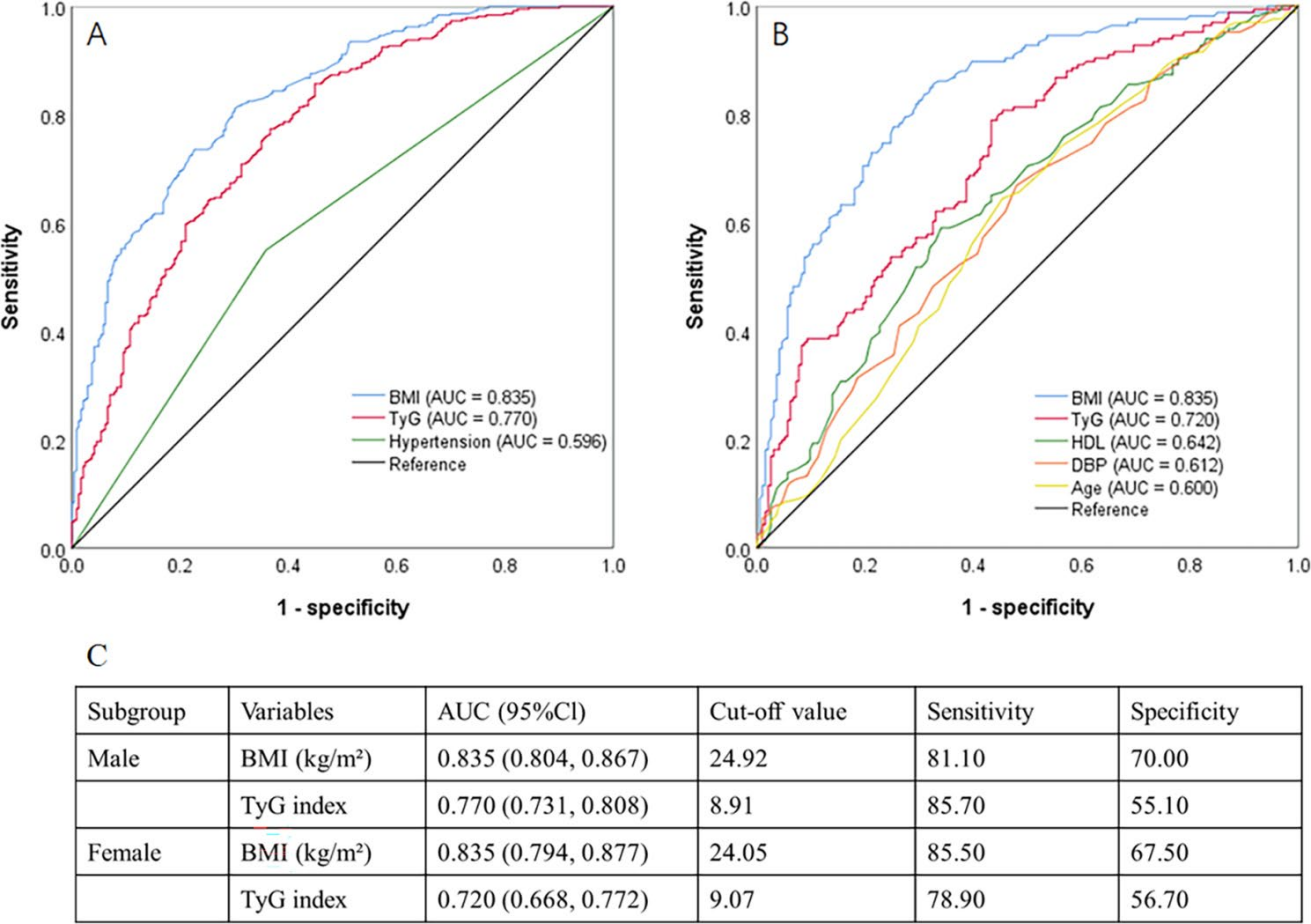
ROC analyses of independent factors for VO. **(A)** ROC analyses in male patients; **(B)** ROC analyses in female patients; **(C)** Cut-off values of BMI and TyG index in male and female patients

Logistic regression analysis demonstrated that BMI and TyG index were independent factors associated with VO in both males (odds ratio [OR] = 1.580 and 2.997, respectively) and females (OR = 1.589 and 2.233, respectively) (Fig. 3). For the prediction of VO, both BMI and TyG index had acceptable powers in male (AUC = 0.835 and 0.770, respectively) and female patients (AUC = 0.835 and 0.720, respectively) (Fig. 4). In male and female patients, the cut-off values of BMI were 24.92 kg/m^2^ and 24.05 kg/m^2^, and of TyG index were 8.91 and 9.07, respectively (Fig. 4).

**Fig. 5:**
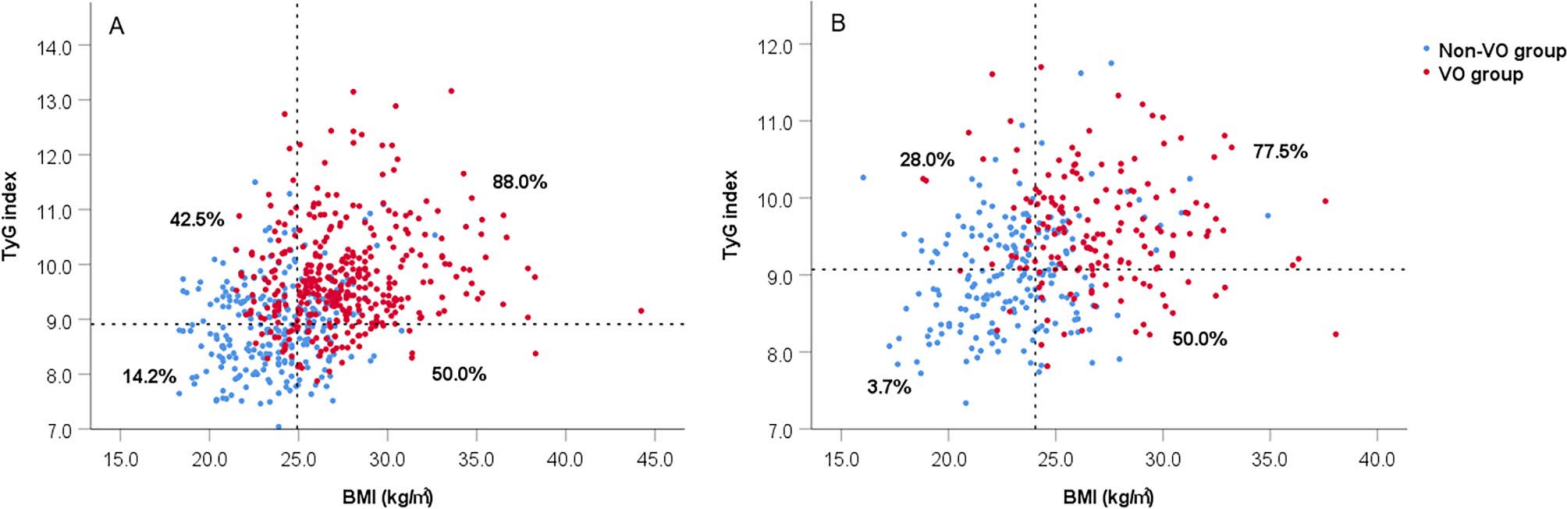
Scatter diagram of the distribution of VO and non-VO patients according to their BMI and TyG index. **(A)** male patients; **(B) **female patients

The scatter plot demonstrates the distribution of VO and non-VO patients according to their BMI and TyG index (Fig. 5). The patients were classified according to the cutoff values of BMI and TyG index. Patients with higher values of both BMI and TyG index showed a significantly higher risk of VO than the other patients. The adipose tissue and clinical characteristics of patients with different BMI and TyG index levels were shown in Supplemental Tables. The patients with higher BMI and TyG index levels had more severe disorders of glucolipid metabolism and adipose distribution.

**Fig. 6:**
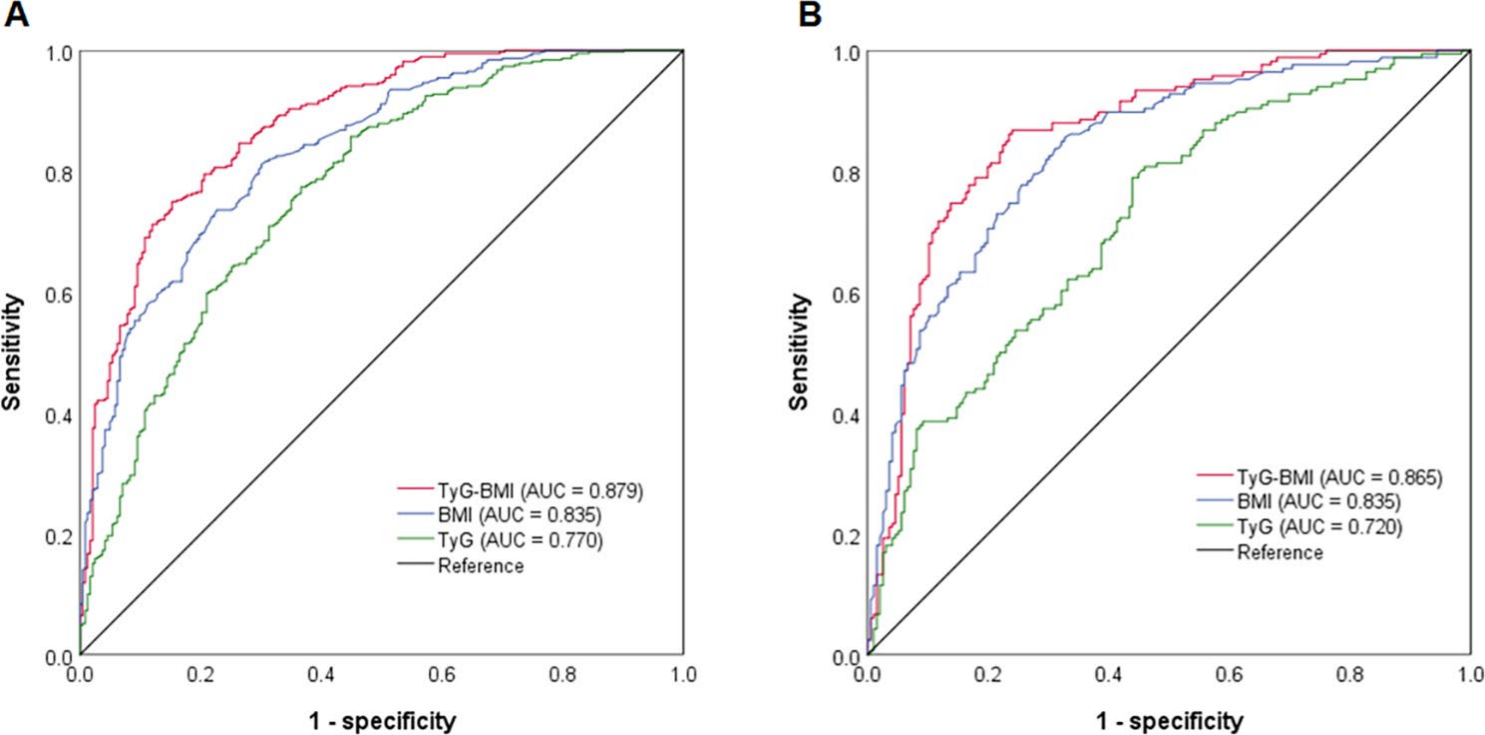
ROC analyses of TyG-BMI for VO.**(A)** male patients; **(B)** female patients

As shown in Fig. 6, TyG-BMI, the combination index of TyG and BMI, showed significantly higher predictive power (AUC = 0.879) than BMI (AUC = 0.835) and TyG (AUC = 0.770) for VO in male patients. In female patients, TyG-BMI also showed significantly higher predictive power (AUC = 0.865) than TyG (AUC = 0.720) but showed no significance when compared with BMI (AUC = 0.835).

### Addition analysis of WC and WHR in partial patients

Only 218 patients (125 men and 93 women) have the recorder of WC and WHR. Among all variables, WC showed the highest correlation with VAA in males (r = 0.746) and females (r = 0.716). As shown in Supplemental Fig. 1, WC showed strong predictive power for VO in males (AUC = 0.831) and females (AUC = 0.896). TyG-WC, the combination index of TyG and WC, showed higher diagnostic performance for VO in males (AUC = 0.860) but not in females (AUC = 0.890).

## Discussion

In this study, we demonstrated an association between VO and the TyG index in patients with T2DM. The TyG index is a more efficient predictor of VO in individuals with T2DM than other biochemical indicators. The statistical cut-off value of the TyG index was 8.91 in males and 9.07 in females for VO prediction. These findings are clinically relevant because VO is an important health issue that needs to be detected early. However, current equipment is unsuitable for screening at the population level in primary care. According to our study, patients with higher values of both BMI (＞24.92 kg/m^2^ in males, ＞24.05 kg/m^2^ in females, respectively) and TyG index (＞8.91 in males, ＞9.07 in females, respectively) are more likely to have VO, which means that further examination is warranted.

Several anthropometric indices for visceral fat have been developed, such as BMI, WC, and WHR [26]. As biochemical indicators were not considered, these anthropometric indices alone cannot adequately assess central abdominal fat mass. They do not account for the extreme variation in visceral fat distribution between individuals [27]. At a given BMI or WC, Asians are characterized by a greater amount of VAT than Europeans [28]. Given the frequent coexistence of excess visceral and liver fat, the so-called hypertriglyceridemic waist phenotype (high levels of TG and WC) has been developed as a tool to screen for the presence of excess VAT [29–31]. However, whether TG is the best indicator for predicting VO combined with WC or BMI has not been explored. Our study demonstrated that the TyG index has a more powerful predictive ability for VO than any other biochemical index.

In studies in Asians, TyG index-related parameters, including TyG-BMI, TyG-WC and TyG-WHR, showed higher predictive power for non-alcoholic fatty liver disease [20, 21]. Given the possibility that fatty liver reflects insulin resistance, we compared the overall predictive power of TyG-BMI with BMI. Our study demonstrated TyG-BMI had a higher diagnostic performance for VO than BMI in patients with T2DM. Therefore, TyG, combined with BMI, can improve the early diagnosis of VO and help the clinician develop more appropriate interventions to prevent or delay metabolic complications.

The distribution of adipose tissue also plays a crucial role in the progression of T2DM and its vascular complications [32]. Visceral and ectopic adiposity confer a much higher risk of cardiovascular disease than subcutaneous adiposity [33]. Large cohort studies have documented excess VAT as a predictor of the development of cardiovascular risk factors over time, independent of total body fat mass or subcutaneous adipose tissue levels [34, 35]. Lower abdominal fat density is also associated with the profile of biomarkers and may be a valid biomarker of cardiometabolic risk [13]. In some studies, VSR has been reported to be more strongly associated with cardiometabolic risks than VAA [36–40]. Therefore, except VAA, both VSR and VAD are also important indicators for cardiometabolic diseases. The present study demonstrated that the TyG index is significantly correlated with VAD and has a stronger correlation with VSR than BMI, which has also been confirmed in the PREDIMED-Plus trial [41]. This evidence indicated that the TyG index is a more comprehensive indicator of adipose volume, density, and distribution.

Several studies have confirmed the association between the TyG index and metabolic diseases such as metabolic syndrome, diabetes, nonalcoholic fatty liver disease (NAFLD), and cardiovascular disease [42–47]. The cut-off values of the TyG index were 8.7 for the prevalence of metabolic syndrome [42], 8.5 for NAFLD [47], and 9.3 for major adverse cardiovascular events in patients with diabetes [43]. These values are close to 9 and are consistent with the cut-off values of VO in this study. The consistency of the cutoff values for the TyG index in predicting these metabolic diseases may be attributed to the insulin resistance induced by VO.

Several pieces of evidence have suggested strong sex specificity for regional adipose tissue distributions, with females having a higher proportion of gluteal-femoral body (subcutaneous) fat; however, males have more in the abdominal (visceral) region [48, 49]. Similar to the above studies, our study also observed that males have a much higher level of visceral adipose, and females have more subcutaneous fat. This obvious difference in the adipose tissue phenotype according to sex is partially because of sex hormones and genetic variations [50].

This study has several limitations. First, more than 80% of the patients were under antidiabetic treatment, nearly 20% of the patients received statins, and a few subjects took fibrates, which inevitably affected the stability of our results. Second, we continuously collected data from all participants at a particular location over time; thus, our participants represent hospitalized patients with T2DM but not the general population with T2DM. Third, only 25% of included patients had a record of WC and WHR. The predictive ability of TyG, in combination with these anthropometric indices for VO, needs to be confirmed by further large sample studies. Fourth, the predictive ability of HOMA-IR for VO was not compared because serum C-peptide and insulin were susceptible to antidiabetic drugs and not routine inspection items in our hospital. Finally, because our study was conducted in Chinese adults with T2DM, the findings may not be readily generalizable to other populations or ethnicities.

In conclusion, we showed a significant association between an increased TyG index and VO in patients with T2DM. The TyG index is a comprehensive indicator of adipose volume, density, and distribution in patients with T2DM. It is a valuable predictor of VO in combination with anthropometric indices, such as BMI.

## Electronic supplementary material

Below is the link to the electronic supplementary material.

Supplemental Table 1. The adipose tissue and clinical characteristics of male patients with different levels of BMI and TyG index
Supplemental Table 2. The adipose tissue and clinical characteristics of female patients with different levels of BMI and TyG index
Supplemental Fig. 1. ROC analyses of TyG-WC and TyG-WHR for VO (A) male patients; (B) female patients

## Data Availability

All data generated or analyzed during this study are included in this published article. Further inquiries for the original data can be directed to the corresponding authors.

## Abbreviations

ALT: Alanine transaminase
ALB: Albumin
AUC: Area under the ROC curve
BUN: Blood urea nitrogen
BMI: Body mass index
CT: Computed tomography
Cr: Creatinine
DBP: Diastolic blood pressure
FBG: Fasting blood glucose
FT4: Free thyroxine
FT3: Free triiodothyronine
HbA1c: Glycated hemoglobin
Hb: Hemoglobin
HbA1c: Glycated hemoglobin
HDL: High-density lipoproteins
LDL: Low-density lipoproteins
NAFLD: Nonalcoholic fatty liver disease
OR: Odds ratio
ROC: Receiver operating characteristic
SAA: Subcutaneous adipose area
SAD: Subcutaneous adipose density
SAT: Subcutaneous adipose tissue
SBP: Systolic blood pressure
TSH: Thyroid-stimulating hormone
TC: Total cholesterol
TG: Triglyceride
TyG: Triglyceride glucose
T2DM: Type 2 diabetes mellitus
VSR: VAA-to-SAA ratio
VAA: Visceral adipose area
VAD: Visceral adipose density
VAT: Visceral adipose tissue
VO: Visceral obesity
WC: Waist circumference
WHR: Waist hip rate
