# Supplementary material for: Serum triglyceride glucose index is a valuable predictor for visceral obesity in patients with type 2 diabetes: a cross-sectional study
Source: Cardiovasc Diabetol. 2023 Apr 29;22:98. doi: 10.1186/s12933-023-01834-3 (PMC10148999; doi:10.1186/s12933-023-01834-3)
Supplement: Supplementary file 1 — Supplemental Table 1. The adipose tissue and clinical characteristics of male patients with different levels of BMI and TyG index Supplemental Table 2. The adipose tissue and clinical characteristics of female patients with different levels of BMI and TyG index Supplemental Fig. 1. ROC analyses of TyG-WC and TyG-WHR for VO (A) male patients; (B) female patients [file 12933_2023_1834_MOESM1_ESM.docx]

**Supplemental Table 1. Adipose tissue and clinical characteristics of male patients with different levels of BMI and TyG index.**

| Variables | BMI-/TyG-  (n = 113) | BMI-/TyG+  (n = 127) | BMI+/TyG-  (n = 74) | BMI+/TyG+  (n = 300) |
| --- | --- | --- | --- | --- |
| Age (years) | 56.73 ± 9.32 ^b, d^ | 53.06 ± 12.20 ^a^ | 54.53 ± 11.67 ^d^ | 51.21 ± 12.37 ^a, c^ |
| DM duration (years) | 9.41 ± 7.35 ^d^ | 7.63 ± 7.36 | 8.95 ± 6.31 ^d^ | 7.24 ± 6.48 ^a, c^ |
| BMI (kg/m^2^) | 22.51 ± 1.77 ^c, d^ | 22.86 ± 1.46 ^c, d^ | 27.03 ± 2.05 ^a, b, d^ | 28.48 ± 2.92 ^a, b, c^ |
| SBP (mmHg) | 134.24 ± 15.57 | 130.98 ± 16.74 ^c, d^ | 136.39 ± 19.06 ^b^ | 137.69 ± 17.59 ^b^ |
| DBP (mmHg) | 80.49 ± 11.46 ^d^ | 80.50 ± 11.88 ^d^ | 81.62 ± 14.19 | 84.01 ± 13.73 ^a, b^ |
| Hemoglobin (g/L) | 141.36 ± 14.40 ^d^ | 145.64 ± 21.60 | 141.28 ± 13.88 ^d^ | 149.52 ± 20.60 ^a, c^ |
| Creatinine (μmol/L) | 65.04 ± 15.10 ^b, d^ | 60.64 ± 14.84 ^a, c, d^ | 68.86 ± 13.74 ^b^ | 68.68 ± 16.45 ^a, b^ |
| Blood urea nitrogen (mmol/L) | 5.63 ± 1.50 | 6.40 ± 4.85 | 5.57 ± 1.23 | 6.00 ± 4.36 |
| TG (mmol/L) | 0.82 (0.62, 1.00) ^b, d^ | 1.64 (1.21, 2.47) ^a, c, d^ | 0.97 (0.79, 1.18) ^b, d^ | 2.13 (1.55, 3.31) ^a, b, c^ |
| Total cholesterol (mmol/L) | 4.64 ± 1.67 | 4.72 ± 2.35 | 4.31 ± 1.16 | 4.55 ± 1.25 |
| HDL (mmol/L) | 1.13 ± 0.41 | 1.13 ± 0.45 | 1.17 ± 0.49 | 1.11 ± 0.35 |
| LDL (mmol/L) | 2.77 ± 1.92 | 2.66 ± 1.08 | 2.57 ± 0.86 | 2.65 ± 0.89 |
| Albumin (g/L) | 44.32 ± 4.17 | 44.32 ± 4.10 | 43.91 ± 4.75 | 43.51 ± 4.81 |
| Alanine transaminase (U/L) | 19.20 (13.85, 34.70) | 21.75 (15.35, 33.28) | 18.85 (14.25, 35.15) | 18.80 (14.00, 26.85) |
| FT3 (pmol/L) | 4.75 ± 1.29 | 4.63 ± 1.53 | 4.51 ± 0.80 | 4.55 ± 1.38 |
| FT4 (pmol/L) | 17.73 ± 10.15 | 16.98 ± 3.31 | 18.47 ± 11.85 | 16.90 ± 6.04 |
| TSH (pmol/L) | 2.27 ± 1.43 | 2.09 ± 1.46 | 2.14 ± 1.63 | 2.30 ± 1.94 |
| HbA1c (%) | 8.49 ± 1.72^d^ | 8.70 ± 2.04^d^ | 8.20 ± 1.89^d^ | 9.21 ± 2.40 ^a, b, c^ |
| FBG (mmol/L) | 9.53 ± 3.54 | 9.45 ± 3.94 | 8.58 ± 3.36^d^ | 9.88 ± 4.23^c^ |
| TyG index | 8.32 ± 0.41 ^b, c, d^ | 9.76 ± 0.69 ^a, c^ | 8.46 ± 0.34 ^a, d^ | 9.89 ± 0.81 ^a, c^ |
| VAA (cm^2^) | 92.61 ± 48.97 ^b, c, d^ | 125.34 ± 59.95 ^a, c, d^ | 161.68 ± 70.03 ^a, b, d^ | 216.31 ± 71.86 ^a, b, c^ |
| SAA (cm^2^) | 88.24 ± 36.58 ^c, d^ | 96.17 ± 32.66 ^c, d^ | 144.17 ± 46.73 ^a, b^ | 158.71 ± 61.50 ^a, b^ |
| VSR | 1.05 ± 0.39 ^b, c, d^ | 1.36 ± 0.67 ^a^ | 1.19 ± 0.54 ^a, d^ | 1.48 ± 0.60 ^a, c^ |
| VAD (HU) | -81.51 ± 9.80 ^b, c, d^ | -86.66 ± 9.26 ^a, c, d^ | -90.04 ± 7.47 ^a, b, d^ | -94.67 ± 4.80 ^a, b, c^ |
| SAD (HU) | -87.74 ± 10.32 ^c, d^ | -90.04 ± 8.66 ^c, d^ | -94.84 ± 5.00 ^a, b, d^ | -95.39 ± 5.11 ^a, b, c^ |
| Cigarette smoking (%) | 57 (50.4) ^d^ | 80 (63.0) | 42 (56.8) | 195 (65.0) ^a^ |
| Alcohol intake (%) | 70 (61.9) ^d^ | 81 (63.8) ^d^ | 43 (58.1) ^d^ | 224 (74.7) ^a, b, c^ |
| Dyslipidemia (%) | 30 (26.5) ^b, d^ | 78 (61.4) ^a, c, d^ | 16 (21.6) ^b, d^ | 229 (76.3) ^a, b, c^ |
| Hypertension (%) | 47 (41.6) ^c, d^ | 38 (29.9) ^c, d^ | 48 (64.9) ^a, b^ | 158 (52.7) ^a, b^ |
| Antidiabetic drugs (%) | 98 (86.7) | 103 (81.1) | 65 (87.8) | 247 (82.3) |
| SGLT-2 inhibitors (%) | 3 (2.7) ^c^ | 4 (3.1) ^c^ | 8 (10.8) ^a, b^ | 20 (6.7) ^a^ |
| GLP-1RAs (%) | 0 (0.0) | 0 (0.0) | 0 (0.0) | 1 (0.3) |
| Insulin (%) | 49 (43.4) | 50 (39.4) | 29 (39.2) | 89 (29.7) |
| Thiazolidinediones (%) | 7 (6.2) | 8 (6.3) | 5 (6.8) | 19 (6.3) |
| Statin drugs (%) | 20 (17.9) | 19 (15.0) | 12 (16.2) | 63 (21.0) |
| Fibrates (%) | 2 (1.8) | 1 (0.8) | 3 (4.1) | 5 (1.7) |
| Antihypertensive drugs (%) | 37 (33.0) | 36 (28.3) | 34 (46.6) | 108 (36.0) |

Abreviations: DM duration, diabetes mellitus duration; SBP, Systolic blood pressure; DBP, Diastolic blood pressure; HDL, high-density lipoproteins; LDL, low-density lipoproteins; FT3, free triiodothyronine; FT4, free thyroxine; TSH, thyroid-stimulating hormone; HbA1c, glycated hemoglobin. BMI-, BMI <24.92 kg/m^2^; BMI+, BMI ≥24.92 kg/m^2^ ; TyG-, TyG <8.91; TyG+, TyG ≥8.91. ^a^, p<0.05 vs. BMI-/TyG- group; ^b^, p<0.05 vs. BMI-/TyG+ group; ^c^, p<0.05 vs. BMI+/TyG- group; ^d^, p<0.05 vs. BMI+/TyG+ group.

**Supplemental Table 2. Adipose tissue and clinical characteristics of female patients with different levels of BMI and TyG index.**

| Variables | BMI-/TyG-  (n = 81) | BMI-/TyG+  (n = 74) | BMI+/TyG-  (n = 64) | BMI+/TyG+  (n = 142) |
| --- | --- | --- | --- | --- |
| Age (years) | 57.48 ± 11.38 | 59.05 ± 9.44 | 58.28 ± 9.95 | 59.67 ± 10.02 |
| DM Duration (years) | 11.08 ± 7.83 | 9.66 ± 7.23 | 9.56 ± 7.36 | 9.16 ± 7.21 |
| BMI (kg/m^2^) | 21.43 ± 1.70 ^b, c, d^ | 22.13 ± 1.69 ^a, c, d^ | 26.64 ± 2.59 ^a, b^ | 27.51 ± 2.78 ^a, b, c^ |
| SBP (mmHg) | 129.78 ± 20.05 ^c, d^ | 136.59 ± 24.07 | 138.48 ± 21.96 ^a^ | 138.99 ± 18.68 ^a^ |
| DBP (mmHg) | 73.79 ± 11.17 ^d^ | 77.19 ± 13.90 | 77.23 ± 10.70 | 79.15 ± 11.86 ^a^ |
| Hemoglobin (g/L) | 122.43 ± 18.80 ^b, d^ | 128.89 ± 19.78 ^a^ | 122.27 ± 25.03 | 132.43 ± 20.58 ^a, c^ |
| Creatinine (μmol/L) | 48.60 ± 10.66 | 52.74 ± 18.31 | 51.06 ± 10.84 | 51.10 ± 13.01 |
| Blood urea nitrogen (mmol/L) | 4.95 ± 1.36 | 5.35 ± 1.60 | 6.97 ± 14.66 | 5.29 ± 1.25 |
| TG (mmol/L) | 0.85 (0.70, 1.22) ^b, d^ | 1.59 (1.15, 2.14) ^a, c, d^ | 1.07 (0.91,1.35) ^b, d^ | 1.95 (1.39,2.84) ^a, b, c^ |
| Total cholesterol (mmol/L) | 4.48 ± 0.96 ^b, d^ | 5.22 ± 1.12 ^a, c^ | 4.40 ± 0.87 ^b^ | 5.23 ± 1.36 ^a, c^ |
| HDL (mmol/L) | 1.34 ± 0.29 ^c, d^ | 1.30 ± 0.31^d^ | 1.22 ± 0.24 ^a^ | 1.16 ± 0.26 ^a, b^ |
| LDL (mmol/L) | 2.73 ± 0.82 ^b, d^ | 3.09 ± 0.99 ^a, c^ | 2.74 ± 0.80 ^b^ | 3.09 ± 0.92 ^a, c^ |
| Albumin (g/L) | 42.07 ± 4.83 ^d^ | 43.12 ± 4.93 | 41.89 ± 3.75 | 43.58 ± 4.47 ^a, c^ |
| Alanine transaminase (U/L) | 14.60 (10.95, 19.25) ^d^ | 15.50 (10.95, 41.75) | 15.10 (12.50, 21.30) | 17.10 (12.65, 27.25) ^a^ |
| FT3 (pmol/L) | 4.38 ± 1.68 ^b^ | 3.95 ± 0.73 ^a, c^ | 4.44 ± 1.53 ^b^ | 4.14 ± 0.73 |
| FT4 (pmol/L) | 15.19 ± 3.52 | 16.05 ± 2.73 ^c^ | 14.89 ± 3.70 ^b^ | 15.96 ± 3.06 ^c^ |
| TSH (pmol/L) | 2.58 ± 2.21 | 2.40 ± 1.72 | 2.61 ± 1.45 | 3.22 ± 4.92 |
| HbA1c (%) | 8.04 ± 1.81 ^b, d^ | 9.99 ± 2.24 ^a, c^ | 8.37 ± 1.44 ^b^ | 9.87 ± 2.35 ^a, c^ |
| FBG (mmol/L) | 7.22 ± 2.36 ^b, d^ | 12.92 ± 4.28 ^a, c, d^ | 6.56 ± 1.95 ^b^ | 11.72 ± 4.03 ^a, b, c^ |
| TyG index | 8.48 ± 0.37 ^b, d^ | 9.67 ± 0.51 ^a, c^ | 8.59 ± 0.35 ^b^ | 9.82 ± 0.57 ^a, c^ |
| VAA (cm^2^) | 65.76 ± 29.62 ^b, c, d^ | 96.00 ± 45.37 ^a, c, d^ | 123.30 ± 54.30 ^a, b^ | 152.20 ± 51.30 ^a, b, c^ |
| SAA (cm^2^) | 112.37 ± 40.26 ^c, d^ | 118.83 ± 36.18 ^c, d^ | 175.47 ± 58.25 ^a, b^ | 185.38 ± 59.04 ^a, b^ |
| VSR | 0.62 ± 0.31 ^b, c, d^ | 0.86 ± 0.47 ^a^ | 0.74 ± 0.34 ^a^ | 0.89 ± 0.41 ^a, c^ |
| VAD (HU) | -83.01 ± 7.77 ^b, c, d^ | -87.13 ± 9.15 ^a, c, d^ | -93.28 ± 9.43 ^a, b^ | -93.66 ± 4.56 ^a, b^ |
| SAD (HU) | -94.43 ± 6.97 ^c, d^ | -96.21 ± 6.00 ^c, d^ | -100.41 ± 6.04 ^a, b^ | -98.88 ± 4.87 ^a, b^ |
| Cigarette smoking (%) | 0 (0.0) | 2 (2.7) | 1 (1.6) | 5 (3.5) |
| Alcohol intake (%) | 0 (0.0) | 1 (1.3) | 1 (1.6) | 1 (0.7) |
| Dyslipidemia (%) | 32 (39.5) ^b, d^ | 44 (58.7) ^a, c, d^ | 26 (40.6) ^b, d^ | 108 (76.6) ^a, b, c^ |
| Hypertension (%) | 23 (28.4) ^c, d^ | 31 (41.3) ^d^ | 32 (50.0) ^a^ | 79 (55.6) ^a, b^ |
| Antidiabetic drugs (%) | 76 (93.8) | 67 (89.3) | 61 (95.3) | 129 (90.8) |
| SGLT-2 inhibitors (%) | 5 (6.2) | 3 (4.0) | 5 (7.8) | 7 (4.9) |
| GLP-1RAs (%) | 0 (0.0) | 0 (0.0) | 0 (0.0) | 1 (0.7) |
| Insulin (%) | 41 (50.6) | 37 (49.3) | 30 (46.9) | 65 (45.8) |
| Thiazolidinediones (%) | 2 (2.5) ^b, c^ | 12 (16.0) ^a^ | 7 (10.9) ^a^ | 10 (7.0) |
| Statin drugs (%) | 17 (21.0) | 14 (18.9) | 16 (25.0) | 31 (21.8) |
| Fibrates (%) | 1 (1.2) | 1 (1.3) | 2 (3.1) | 5 (3.5) |
| Antihypertensive drugs (%) | 17 (21.0) ^c, d^ | 19 (25.3) ^c, d^ | 28 (43.8) ^a, b^ | 63 (44.4) ^a, b^ |

Abreviations: DM duration, diabetes mellitus duration; SBP, Systolic blood pressure; DBP, Diastolic blood pressure; HDL, high-density lipoproteins; LDL, low-density lipoproteins; FT3, free triiodothyronine; FT4, free thyroxine; TSH, thyroid-stimulating hormone; HbA1c, glycated hemoglobin. BMI-, BMI <24.05 kg/m^2^; BMI+, BMI ≥24.05 kg/m^2^ ; TyG-, TyG <9.07; TyG+, TyG ≥9.07. ^a^, p<0.05 vs. BMI-/TyG- group; ^b^, p<0.05 vs. BMI-/TyG+ group; ^c^, p<0.05 vs. BMI+/TyG- group; ^d^, p<0.05 vs. BMI+/TyG+ group.


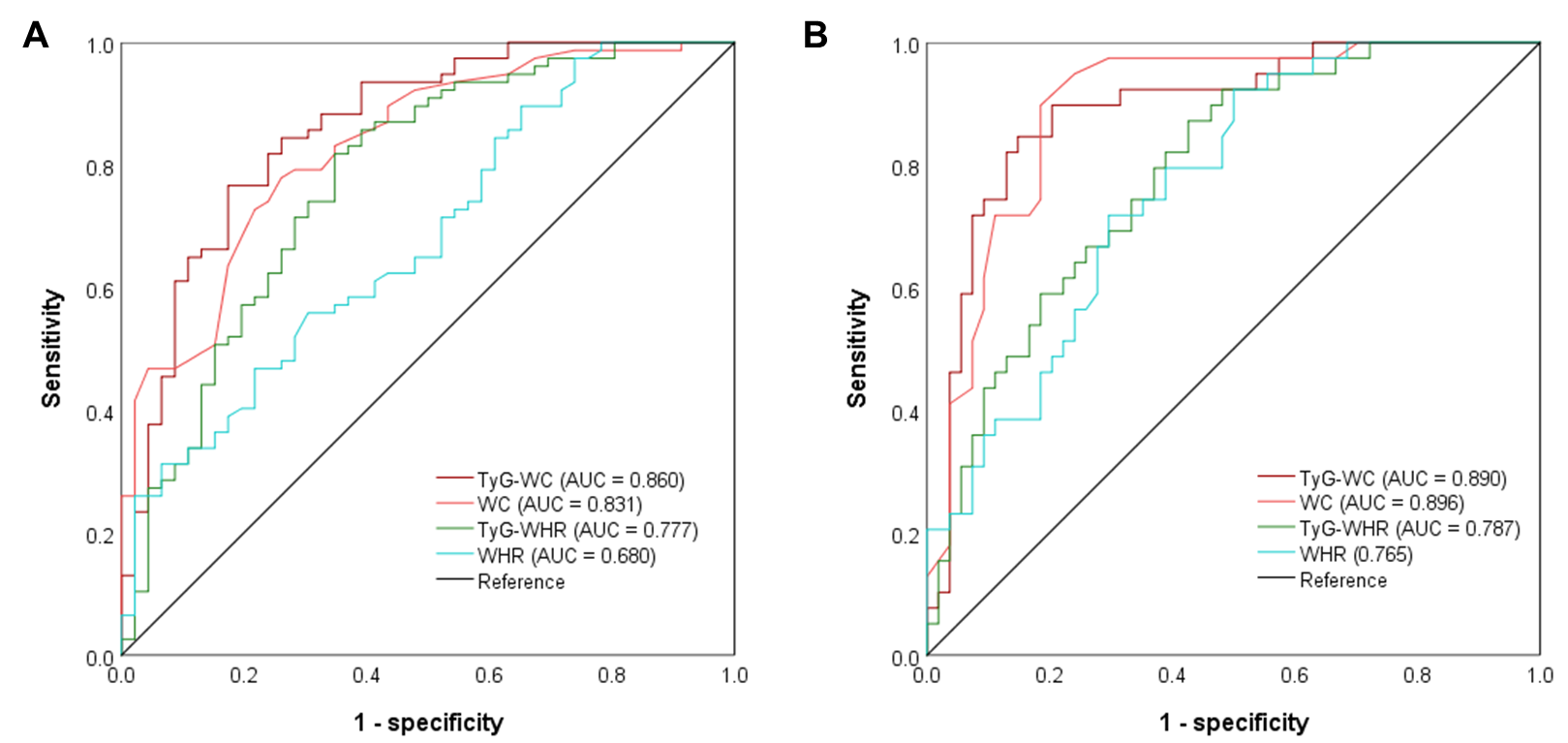


**Supplemental Figure 1. ROC analyses of TyG-WC and TyG-WHR for VO.** (A) male patients; (B) female patients.
